# An Evidence Theory Based Embedding Model for the Management of Smart Water Environments

**DOI:** 10.3390/s23104672

**Published:** 2023-05-11

**Authors:** Maha Driss, Wadii Boulila, Haithem Mezni, Mokhtar Sellami, Safa Ben Atitallah, Nouf Alharbi

**Affiliations:** 1 Security Engineering Lab, CCIS, Prince Sultan University, Riyadh 12435, Saudi Arabia; 2 RIADI Laboratory, University of Manouba, Manouba 2010, Tunisia; mokhtar.sellami@isetj.rnu.tn (M.S.); safa.benatitallah@ensi-uma.tn (S.B.A.); 3 Robotics and Internet-of-Things Laboratory, Prince Sultan University, Riyadh 12435, Saudi Arabia; 4 College of Computer Science and Engineering, Taibah University, Madinah 42353, Saudi Arabia; hmezni@taibahu.edu.sa (H.M.); nmoharbi@taibahu.edu.sa (N.A.); 5 SMART Lab, Jendouba University, Jendouba 8189, Tunisia

**Keywords:** smart water environments, water information network, network representation learning, uncertainty modeling, water monitoring, sensor cloud services

## Abstract

Having access to safe water and using it properly is crucial for human well-being, sustainable development, and environmental conservation. Nonetheless, the increasing disparity between human demands and natural freshwater resources is causing water scarcity, negatively impacting agricultural and industrial efficiency, and giving rise to numerous social and economic issues. Understanding and managing the causes of water scarcity and water quality degradation are essential steps toward more sustainable water management and use. In this context, continuous Internet of Things (IoT)-based water measurements are becoming increasingly crucial in environmental monitoring. However, these measurements are plagued by uncertainty issues that, if not handled correctly, can introduce bias and inaccuracy into our analysis, decision-making processes, and results. To cope with uncertainty issues related to sensed water data, we propose combining network representation learning with uncertainty handling methods to ensure rigorous and efficient modeling management of water resources. The proposed approach involves accounting for uncertainties in the water information system by leveraging probabilistic techniques and network representation learning. It creates a probabilistic embedding of the network, enabling the classification of uncertain representations of water information entities, and applies evidence theory to enable decision making that is aware of uncertainties, ultimately choosing appropriate management strategies for affected water areas.

## 1. Introduction

Water management influences many aspects of our life, including the environment, food production, irrigation, energy generation, etc. [1]. One of the world’s most pressing challenges is the scarcity of safe water, which is quickly dwindling due to climate change, contamination, and pollution. With the explosive rise in the world’s population, the necessity for efficient and smart water resource monitoring methods is becoming particularly crucial. Smart water monitoring is described as applying various computational approaches to offer users appropriate tools and information for water network supervision, control, analysis, and optimization [2]. Several water management solutions have been developed, implementing the most recent advances in information technology to address this issue, all of which are costly and energy-intensive. Recently, the quest for a smart water management system is gaining traction with the birth of the Internet of Things (IoT) [3,4,5]. IoT technology has risen to prominence in a number of vital sectors in recent years, owing to its enhanced capabilities and competitive benefits [6,7,8]. The IoT allows the gathering and analyzing of data in its environment, thus offering intelligent applications in a wide range of areas, notably for water management. The IoT, in this context, refers to a network of sensing devices that gather and monitor water data, which are then transmitted to computing systems for analysis. IoT-based water management systems are low-cost and energy-efficient solutions that can be easily expanded while allowing effective remote monitoring and control [4].

In this context, an IoT-centered solution is extremely advantageous since it enables the control of water quality and the optimization of safe water usage through the use of intelligent corrective actions and policies. However, one possible research direction for additional investigation to improve the effectiveness of this solution is to investigate how to deal with uncertainty when confronted with inaccurate or erroneous water data. Uncertainty is a pervasive feature in real-world scenarios and significantly impacts the quality of the information we can gather from data [9]. In many applications, such as water resource management, the presence of uncertainty can lead to incorrect decisions or suboptimal performance. In fact, multiple sources of uncertainty exist in water environments, which can incorporate bias and inaccuracy into our analysis and decision making if not appropriately addressed. According to [10], various factors contribute to the uncertainty in water data, such as pressure levels, degree of leakage, imprecise calibration of monitoring equipment, and the uncertainties associated with modeling complex water systems. Access to water reserves and flows is frequently challenging and incredibly unpredictable over time and space. Rivers flowing through vegetation or beneath the ice, water moving through porous soil structures and rock fissures, and isolated rainstorms from thunderclouds are just a few examples of these issues. In addition, uncertainty also arises from quantification issues related to errors in water sampling procedures, chemical and biological analyses, water quality indicators, and the assessment of the state of water zones [11]. To ensure reliable and accurate findings, addressing the uncertainty of water data is crucial. This can prevent parameter bias, remove irrelevant data, and enhance water model performance evaluation [10]. Hence, when developing intelligent systems for applications related to water management, it is essential to incorporate methods that can effectively handle and manage the uncertainty of the data.

To address this challenge, we proposed the use of uncertain knowledge graph embedding (UKGE) techniques. These extend the traditional knowledge graph embedding methods by modeling the uncertainty of the data. By incorporating uncertainty into the knowledge graph embedding, we can make more informed decisions by taking into account the uncertainty in the data. For example, in water resource management, UKGE can be used to detect anomalies in the water quality that may be difficult to detect using traditional methods. Additionally, UKGE can improve the performance of downstream tasks, such as classification, clustering, anomaly detection, and link prediction.

In this study, we proposed combining network representation learning with uncertainty handling methods to ensure a rich modeling and efficient management of the water environment. The main contributions of the proposed approach include the following:

- An uncertainty-aware modeling of the smart water environment that quantifies and incorporates uncertainty factors into the water information network (WIN);
- An uncertain embedding of the WIN combining probabilistic and network representation learning (NRL) models to ensure the learning and classification of representations of water information entities under uncertainty of the monitored data;
- An uncertainty-aware decision mechanism that applies the evidence theory, and that consists of querying the uncertain WIN to select the suitable management actions for each class of affected water zones.

The remainder of this paper is organized as follows: In Section 2, we review the current IoT solutions to deal with water management issues. Then in Section 2.2, we briefly present *SmartWater*, our previous sensor cloud-based framework. In Section 3, we discuss the impact of the uncertainty factors on the effectiveness of water management operations. Section 3.1 presents an uncertainty-aware modeling and representation learning of the water information network. It also presents a decision mechanism that exploits the learned representations in triggering appropriate water management plans. Section 4 provides extensive experiments on the proposed approach. The last section is devoted to the conclusion and future work.

## 2. Related Works

### 2.1. Smart Water Management: Recent Related Studies

This section presents relevant and recent studies addressing water scarcity, a global concern caused by various factors such as climate change, pollution, and excessive water consumption. The section underlines the necessity for a real-time water management system to solve this issue and maintain a stable and safe water supply. Furthermore, it emphasizes the potential of future technologies in the realm of water sustainability, such as IoT and cloud computing [4,5,12,13,14].

In [15], the authors aimed to guarantee the proper water resource management for smart cities by proposing a context-aware ontology-driven approach. The proposed system was established on the basis of Multimedia Web Ontology Language (MOWL). The MOWL included three different layers: data collection, context-aware service, and application. The first layer collected data, subsequently translated into a predetermined RDF format in the second layer that produced MOWL files. The final layer ensured that the learned knowledge was presented to the water authority and that the necessary actions were taken in water deficit areas. The authors of [16] utilized a system called FLARE to manage fresh water. This system performed frequent ecological forecasts by utilizing water quality sensors to monitor and regulate water quality in critical lakes and reservoirs. Additionally, FLARE predicted future water quality issues. Cloud computing features were used for remote monitoring and transmission of observational data. In [17], the author proposed an intelligent system for water quality monitoring based on IoT technologies and remote sensors. The approach focused on using remote sensors to measure the four main water quality parameters: pH, temperature, oxidation–reduction potential, and conductivity. The data were transmitted to the cloud, where they were analyzed to perform the appropriate actions. Shahanas et al. [18] presented a Smart Management Water (SMW) system. They began by manually gathering the required dataset, which was then uploaded to a central server through Arduino and Raspberry Pi devices for analysis. The investigation findings were then visualized using a web interface to generate an alert when the water level in a container fell below a predefined limit. The main limitation identified in [15,16,17,18] is the lack of focus on corrective actions. These studies mainly focused on modeling water-related concepts rather than providing effective solutions to the identified issues.

In [19], a Water Quality Management (WQM) system based on a customized intelligent sensor network was presented. This system measured five water parameters, including pH, temperature, carbon dioxide on the surface, turbidity, and water level, using many sensor devices that were monitored simultaneously. The WQM system contributed to smart environmental management by reducing the duration and cost necessary to monitor water quality. Similarly, Mukta et al. [20] proposed a Smart Water Quality Monitoring (SWQM) system to collect the measurements of four water parameters, including pH, turbidity, water temperature, and electric conductivity, using IoT sensors. The SWQM system used a forest classification model to assess the collected measurements and determine whether the water was potable. To demonstrate its efficiency, the performance of this model was compared to other classification methods, including logistic regression, support vector machine, and average perceptron methods. In [21], the authors proposed a water management system based on microservices architecture called WISdoM. Using different data sources, this system combined core functionalities to implement three water utility scenarios, including long-term water demand projections, underground water data management, and water quality monitoring. A microservice encapsulated these data sources and allowed querying the required data. A message broker service was also used to combine different data sources. Expert users assessed the applicability of the suggested approach and the usability of WISdoM by running several scenarios. The authors in these works focused on monitoring water resources without considering the effects of uncertain information gathered throughout IoT sensors.

In the article [22], remote sensing methodologies for measuring irrigation were improved. By integrating remote sensing with soil parameters, the authors were able to accurately model soil water deficit and quantify irrigation water usage for two fields in South Australia. The paper aimed to achieve three goals: (1) to investigate the feasibility of measuring irrigation at the paddock scale using moderate spatial resolution remote sensing observations and soil water deficit modeling; (2) to assess the impact of using different sources of soil properties and conduct an uncertainty analysis of the available parameter values; and (3) to evaluate the potential benefits of using higher spatial and temporal resolution satellite data compared to the moderate resolution Landsat. The study’s goal presented in [23] was to find the best machine learning algorithm with optimal hyperparameters for predicting Water Quality Indices (WQIs) at several monitoring stations in Cork Harbor, Ireland. The study compared eight commonly used ML methods to identify the best models for reducing prediction uncertainty and improving model structure, particularly for coastal WQIs. These studies were limited in that they did not investigate water quality/quantity in terms of time resolution. Additional investigation is needed to determine how effectively various techniques predict WQIs and water resources’ levels utilizing data attributes that change over time. This will allow for an improved understanding of the temporal variations of water quality/quantity and a more accurate forecast.

After conducting a thorough analysis of the aforementioned studies, we have identified several further concerns:

- *Lack of standardization and resource constraints:* There is a lack of standardization in the methods used for monitoring and managing water environments, making it difficult to compare data from different sources or develop consistent models for decision-making. Furthermore, the resources available for monitoring and managing water environments may be limited, affecting the quality and quantity of collected data and the ability to make decisions based on that data. To provide a standard method to model, monitor, and manage water environments and solve problems related to resource limitations in terms of water quality and quantity management, we represent the water environment as a knowledge graph, which identifies the elements involved, such as water entities, sensors, water issues, observed data, water management processes, and so on. This multi-relational and semantic structure serves as a dictionary, including all water-related information. In addition, we exploit network embedding to progressively acquire semantics and rich representations of water entities and transfer them into a low-dimensional vector space based on their related characteristics, behaviors, and variations. This stage aids in the classification of impacted water entities as well as the efficient selection and execution of relevant corrective actions.
- *Uncertainty of water environments:* Current solutions failed to represent correctly and model uncertainty factors and sources, as they assume sensors correctly capture the monitored data. However, water environments are ever-changing by nature, and their sensor infrastructure is often subject to unstable behavior, leading to inaccurate or incompleteness of collected data. We solve this issue by modeling and quantifying uncertainty at different levels of the water information network, including the water entities level and the management policies level.
- *Water network complexity:* As complex water information networks are processed in a real-time and continuous way, such a graph-like structure coupled with uncertainty sources is expected to add a new complexity factor. To solve this issue, we extend our previous incremental embedding model [24] by incorporating confidence scores into the factual relations between the nodes (water entities, events, management policies).
- *Decision making granularity:* Current approaches to water management, including smart solutions, perform management operations at a high granularity level and in an isolated manner. However, in the water network, several entities may feature similar deviations (e.g., pressure loss) and require compatible management policies (e.g., specific discharge level to the canal). To ensure efficient handling of water zone issues, we precede the decision process by classifying those entities while considering the confidence of their related knowledge and the monitoring step’s output.

### 2.2. Our Previous Work

Aiming to provide a water management solution in smart environments, we proposed in [24] a sensor cloud-based four-layer framework that takes advantage of a cloud of water sensors that are distributed across multiple water zones. The collected data were processed at the data management layer. Then at the workflow and water analytics layer, corrected measures are triggered for each class of detected problem.

#### 2.2.1. Water Information Network Modeling and Embedding

To ensure efficient management of water zones, we adopted a knowledge graph [25] modeling of water zones’ major entities (e.g., pipelines, reservoirs, water deviations, management policies). This graph-like structure (see Definition 1), initially introduced by Google, offered a multi-relational, multi-source, and semantic characterization of water entities. The water information network, as defined in [24], consists of the following.

**Definition 1.**  *A Water Information Network is defined as a diverse information network $G=(V,E,F,D^{+})$. Here, $V=<V_{s},V_{c},V_{f},V_{z}>$ refers to the collection of water-related entities such as sensors, services, water zones, management policies, etc. The edges E in the network correspond to the connections between the different entities present in the water environment. The set F denotes the features that describe the entities in the water network. $D^{+}=\left(e_{i},r,e_{j}\right)$ represents the set of facts (triples) in G. A fact is a 3-tuple $f=(v_{i},r,v_{j})$ where $v_{i},v_{j}\in V$ correspond to the head and tail entities (e.g., sensors, monitoring hubs, distribution pipelines, management rules, etc.), and r∈E indicates the relation (connection) between $v_{i}$ and $v_{j}$. The relation $r:v_{i}\overset{r}{\to }v_{j}\in E$ is a typed link (e.g., Monitor, ManagedBy, Trigger) that connects the entities $v_{i}$ and $v_{j}$ in the Water Knowledge Graph. This definition is denoted by Definition 1.*

In this paper, we followed a meta path-based embedding of the water network entities, which allowed mapping the water entities with similar features (e.g., distribution pipelines with abnormal behavior, reservoirs with non-drinkable water, etc.) and states as close as possible in the vector space.

The proposed water network embedding model was also implemented in its incremental version to cope with the changes that affect the water network after each detected change. The product metapath2vec [26], an incremental embedding technique suitable for dynamic and heterogeneous information networks, was applied to the updated water information network to adjust the water entities’ distributions and proximity in the embedding vector space. The incremental embedding process instantiates the skip-gram model, which was preceded by a guided random walk that takes as input a set $V^{\prime }=V+\Delta V$ of the water entities affected by changes (e.g., leakage, pipeline removal).

#### 2.2.2. Classification-Based Water Management

Since monitoring is the first step towards the effective management of water zones, we designed a sensor cloud-based architecture to collect useful data about water quality and used it to update the water information network. Being subject to continuous and frequent changes, this required re-embedding its entities (e.g., water pipelines, reservoirs) to preserve a correct representation of the water network. For this purpose, meta path-based incremental embedding was applied to cope with the water network complexity and its highly dynamic nature. Furthermore, knowing that several water zones may encounter similar deviations, their vector representation tends to be close in the embedding space. Based on this fact, we have chosen to arrange the vector embeddings into classes of water entities according to their new states [24]. That was the case of water stations, which were classified into poor, good, or excellent quality zones. Finally, based on the classified embeddings, a corrective measure was triggered for each class of problem (e.g., leakage, pressure loss, chlorination), rather than selecting a management policy for each separate water zone.

### 2.3. Motivations

Based on the identified drawbacks (see Section 2.1) and the challenges related to our previous work (see Section 2.2), we identified several differences between the present research and state-of-the-art approaches (see Table 1).

In addition to the above differences, the use of network representation learning (NRL) has been proven as a useful method for dealing with the constantly changing water network [27,28]. NRL involves converting the network structure into a vectorized form, which enables downstream tasks such as clustering, classification, anomaly detection, and recommendation to be performed on the learned vector representations of node features [12]. NRL is an effective means of handling graph-like structures and extracting valuable information through various downstream tasks, such as classification, which is the focus of this work. Several arguments support the effectiveness of NRL as a technique for embedding modeling and classification in a water environment:

- *NRL captures complex relationships:* NRL can capture complex relationships between nodes in a water network, which can be difficult to model using traditional techniques. By representing the network as a vectorized form, NRL can preserve the structural information of the network, including its topology, connectivity, and node attributes.
- *NRL handles changing network structure:* Water networks are constantly changing due to changes in water demand, pipe breaks, and other factors. NRL effectively handles these changes by updating the network representation as the structure evolves. This ensures that the learned representations remain up-to-date and accurate.
- *NRL handles large-scale networks:* Water networks can be vast, consisting of thousands or even millions of nodes. NRL can efficiently handle large-scale networks by leveraging artificial-intelligence-based techniques, which can scale up to large graphs while preserving the structural information of the network.
- *NRL is used for various downstream tasks.* This makes it a versatile technique for analyzing, modeling, and processing water networks.

In our work, the proposed embedding model learns water entities’ representations according to the confidence score (truth degree) of various pieces of data. The probabilistic embedding of the water information network effectively exploits the uncertainty of water-related data, allowing a more accurate prediction of their quality. We followed a probabilistic and uncertain embedding logic to approximate these uncertainties and provide correct recommendations.

Probabilistic models have gained widespread acceptance in different domains, particularly recommender systems. Incorporating such models (e.g., latent probabilistic models, latent Dirichlet allocation, probabilistic matrix factorization, probability relevance, and probability ranking principles) to decision support systems has been a promising approach. Probabilistic knowledge graph embedding has been applied in some domain-independent approaches [28,29]. However, this technique has not yet been exploited in water management.

## 3. Uncertainty Handling in Water Environments

Despite the dramatically increasing number of water monitoring approaches, most ignore the uncertainty factors (e.g., pressure level, leakage degree, imprecise calibration of monitoring equipment, uncertainties associated with the modeling of complex water systems, inaccurate sensing, incorrect or incorrect or missing measurements, etc.). Such uncertainty must be considered during the water monitoring and network embedding process. Uncertainty is a natural feature of many forms of knowledge. In real-world uncertain knowledge graphs such as ConceptNet, NELL, and ProBase, relations and facts are associated with a confidence score [30]. Currently, there are few alternatives to capture uncertainty information with knowledge graph embeddings [28,29]. To achieve the goal of water monitoring under uncertain water zones’ contexts, it is important to encode additional information (e.g., truth degrees of water measurements) to preserve uncertainty. Probabilistic models have gained widespread acceptance in different domains, particularly recommender systems [31,32,33,34,35]. Probabilistic knowledge graph embedding has also been applied in some domain-independent approaches [28,29]. However, uncertain and probabilistic embedding have not yet been exploited in the field of water monitoring. Therefore, incorporating such models (e.g., latent probabilistic models, latent Dirichlet allocation, probabilistic matrix factorization, probability relevance, and probability ranking principles) to water monitoring systems would be a promising approach.

The present work aims at improving our smart water monitoring system by incorporating uncertainty into the monitoring process. An uncertain water information network, also called UWIN (see Section 3.1), will represent knowledge as a set of facts denoting the contextual relations defined over water entities. The UWIN will also contain uncertain facts and will provide a confidence score, along with each contextual relation between water entities and sensors. This approach considers the UWIN as a set of probabilistic facts. Each relation between two entities in UWIN (e.g., reservoir, sensor, pipeline, etc.) is represented with a probability value. The probabilistic construction of the UWIN effectively addresses the uncertainty of water zones’ information, allowing for a more accurate prediction of their states. We will adopt a probabilistic graph embedding method to approximate these probabilities and provide recommendations for the appropriate water management actions. In this work, we define a model for uncertain knowledge graph embedding to preserve structural relationship information and uncertainty information of contextual relations between water entities in the embedding space. The UWIN model learns the embeddings according to the truth degrees of uncertain contextual relations. A model for uncertain knowledge graph embedding is defined in this work to preserve both structural relationship information and uncertainty information of contextual relations between water entities in the embedding space. The UWIN model learns the embeddings according to the truth degrees of uncertain contextual relations, such as water measurements. In this case, the prediction step consists of forecasting the water quality probability to determine suitable recommendations for actions.

### 3.1. Modeling of Uncertain Embedding of the Water Information Network

#### 3.1.1. Uncertainty Quantification

Water management systems often are subject to uncertainties. Several uncertainty factors may affect the decision quality in a water monitoring system. For example, the uncertainty of input data may be caused by inaccurate measurements, missing values, spatial interpolations, temporal aggregation, assumptions in boundary and initial conditions; or (ii) parameters uncertainty, natural variability, lack/inadequacy of observations, calibration techniques, etc. Monitoring instruments and sensors may also be subject to failures, calibration errors, or unstable behavior, which may affect the monitoring records. That includes the inaccurate measurement of water temperature or turbidity, which is used to determine the clarity of the water, TDO (Total Dissolved Oxygen) and pollution levels, errors in measuring pump rate and pressure, etc. Other important sources of uncertainty concern the insufficient number and geographical spread of sensors, the sampling (i.e., sampling location and frequency), and analytical uncertainties. Hence, an incomplete understanding of the water zones’ states will lead to inappropriate decisions.

The above uncertainty factors and sources must be considered when constructing the water information network (see Section 2.2) and updating it after each monitoring time frame, thus treating it as an uncertain knowledge graph.

The uncertainty related to parameters in the WKG has two forms: aleatory and epistemic. The first refers to a random event’s natural variability, while the second depicts a lack of knowledge. In this paper, uncertainty related to parameters is propagated using belief function theory [36,37]. This theory is effective for modeling and processing aleatory and epistemic uncertainty in a very natural way [38]. To better understand the mechanism of the evidence theory, we will start by explaining the core concepts of this theory, namely the basic belief assignment, uncertain parameters, and propagation of the parameter uncertainty. The main advantages of evidence theory include its ability to handle both aleatory and epistemic uncertainty, its ability to propagate uncertainty in a rigorous and efficient manner, and its ability to incorporate expert knowledge into the uncertainty modeling process. Additionally, evidence theory can provide a measure of the reliability of the results obtained, allowing decision makers to evaluate the level of confidence in the decision-making process. Overall, the use of evidence theory can lead to more accurate and robust decision making in the face of uncertainty.

**Definition** **2.**  *(Basic belief assignment (BBA)) Let $\Theta =\{C_{1},\ldots ,C_{n}\}$ be a finite set of mutually exclusive and exhaustive classes of water quality, called the frame of discernment. A BBA is a function that maps each proposition A from $2^{\Theta }\to \left[0,1\right]$ and verifies that the mapping m(A)≥0, m(∅)=0, and $\sum_{A\in \Theta }m\left(A\right)=1$.*

**Definition** **3.**  *(Uncertain parameters) Epistemic parameters are bounded in a vector $e\in R^{n}$. $e_{i}\left(i\in \left[1,\ldots ,n\right]\right)\to \left[e_{i}^{L},e_{i}^{U}\right]$ having a BPA structure defined as $\left[e_{1}^{L},e_{1}^{U}\right]/m_{1},\ldots ,\left[e_{n}^{L},e_{n}^{U}\right]/m_{n}$. Aleatory parameters $a_{j}\left(j\in \left[1,2,\ldots ,m\right]\right)$ are bounded in a random vector $a_{j}\in R^{m}$ with a normal probability distribution: a∼(μ,σ), where μ is the mean and σ is the standard deviation.*

The belief function theory only considers an interval with an associated mass as input. Therefore, aleatory parameters are transformed into intervals with associated mass values [μ−ξσ,μ+ξσ]. Then, these intervals are discretized into N subintervals [$a_{i}^{L},a_{i}^{U}$], where $m\left(a_{i}\right)=\int_{a_{i}^{L}}^{a_{i}^{U}}f\left(x\right)dx$ and f(x) is the probability density distribution function (pdf) of *x* depicted by Equation (1).

(1) $$f_{N}\left(\mu ,\sigma^{2}\right)\left(x\right)=\frac{1}{\sqrt{2\pi \sigma^{2}}}\;exp\left(-1/2{\left(\frac{x-\mu }{\sigma }\right)}^{2}\right),\;\forall x\in R$$

After computing the BPA structures for the uncertain parameters of the WKG, they will be integrated into a joint structure, and computed as a Cartesian product $c_{ij}=a_{i}\times e_{j}$. The BPA of $c_{ij}$ values are determined according to the following equation, $m\left(c_{ij}\right)=m\left(a_{i}\right)\times m\left(e_{j}\right)$. The responses of the WKG model are estimated as follows $\left[Y_{min},Y_{max}\right]=\left[min_{x\in c_{ij}}f\left(X\right),max_{x\in c_{ij}}f\left(X\right)\right]$.

#### 3.1.2. Water Network Modeling

A first step towards the efficient management of water zones is the accurate monitoring of their state. This task must be preceded by an explicit representation of each water zone’s elements. However, the complexity of the water network coupled with the deviations of sensing objects makes smart monitoring a challenging task. Moreover, sensors may provide incorrect, inaccurate, or incomplete monitoring data, adding a new uncertainty factor regarding the water zones’ state. Seen as an uncertain information network, the present work aims to endow water monitoring systems with uncertainty-handling capabilities. We first model the water information network as an uncertain knowledge graph to achieve this goal. Leading companies have successfully adopted knowledge graph technology (e.g., Facebook, Amazon, Yahoo, etc.), improving service consumers’ quality of experience [39]. However, this new kind of knowledge base does not still support uncertain knowledge, as the multi-relational and valid facts represent semantic modeling of its elements. To solve these issues in the context of smart water monitoring, each relation and feature in the water information network is characterized by a set of values denoting its truth degree. Entities such as water stations, sensors, and management policies are key components of water zones. However, some of them may be characterized by inaccurate information, which leads to a lack of understanding of the water zones’ state.

**Definition** **4.**  *An Uncertain Water Information Network is a heterogeneous graph structure $G=(V,E,F,D^{+},P)$, where nodes in $V=<V_{s},V_{c},V_{f},V_{z}>$ is the set of entities in a water zone (sensors, anomalies, management policies), edges in E denote the relations between the water entities, and the set F represents the features characterizing water entities. $D^{+}=\left\{\left(e_{i},r,e_{j}\right)\right\}$ is the set of weighted/uncertain facts (triples) in G. Each of these is a 4-tuple $f=(v_{i},r,v_{j},l)$, where the heads and tails $v_{i},v_{j}\in V$ correspond to the water network entities (e.g., sensors, anomalies, monitoring hubs, distribution pipelines, management policies), r∈E is a relation between $v_{i}$ and $v_{j}$, and $l=P_{ij}$ is the confidence score (truth degree) denoting the probability that the relation between $v_{i}$ and $v_{j}$ is valid and exists in the UWIN. A confidence score is a value p∈P, where 0≤p≤1. A relation $r:v_{i}\overset{r}{\to }v_{j}\in E$ in the WKG is a typed link (e.g., Monitor, ManagedBy, Trigger) between entities $v_{i}$ and $v_{j}$.*

**Figure 1:**
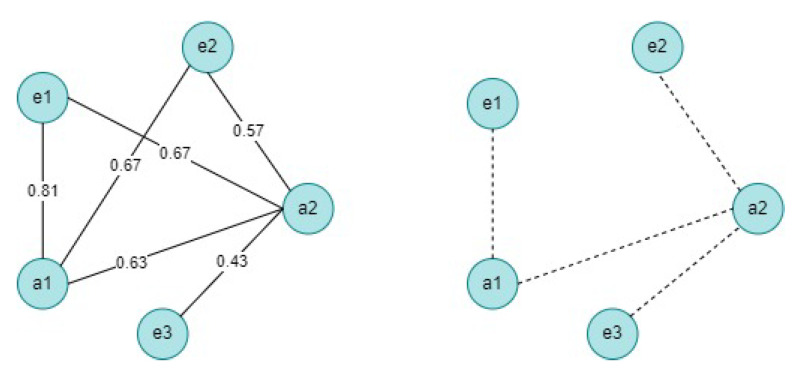
Example of an uncertain water information network (on the left) and possible world (on the right).

In the present work, uncertainty is handled at two levels. At the monitoring level, the collected data could be inaccurate or incorrect (e.g., a range of observed behaviors in a pump station), which requires computing the probability that an observation is true. At the water information network level, a fact’s validity has a truth degree, also called a confidence score. Taking the example of the fact *<Pollution, ManagedBy, SedimentRemoval>*, a high confidence score of this triple (↑1) means a high probability of triggering a sediment removal action in response to detected pollution in a water zone. Contrariwise, a low confidence score (↓0) recommends excluding the sediment removal action from a water management plan. However, the structure of the UWIN at a given time depends on the truth of monitored data. For example, several pH scales could be observed in one water zone (e.g., (7: *pure*, 10: *detergent*, 12: *bleach*)). In such a situation, we have three possible worlds for the UWIN (see Figure 1). In fact, the first scale returned by sensors reflects a pure water state, while the two other scales require triggering a water management plan.

A possible world of a water information network G is a deterministic graph $W=(V,E_{w})$, where $E_{w}\subseteq E$. Hence, given a water zone’s state, the corresponding possible world W is defined by the following probability:

(2) $$P\left(W\right)=\prod_{e\in E_{w}}P_{e}\prod_{e\in E\setminus E_{w}}\left(1-P_{e}\right)$$

Taking the example of the UWIN in Figure 1, the probability of W is computed as follows: $W=\{\left(e_{1},a_{1}\right),\left(e_{1},a_{2}\right),\left(e_{2},a_{2}\right),\left(e_{3},a_{2}\right)\}$ with probability $P\left(W\right)=P_{e_{1}a_{1}}P_{e_{1}a_{2}}P_{e_{2}a_{2}}P_{e_{3}a_{2}}\left(1-P_{a_{1}e_{2}}\right)\left(1-P_{e_{1}a_{2}}\right)=0.81\times 0.67\times 0.57\times 0.43\times 0.33\times 0.33=0.01448$.

To identify the correct triggering situations and to ensure accurate querying of the UWIN, we propose a three-step process that consists of (1) reasoning over the uncertain monitoring data and (2) embedding uncertain facts in the UWIN, and finally, based on a classification of water zones, (3) mapping the most likely observations and facts in the embedding vector space to the suitable corrective measures.

**Figure 2:**
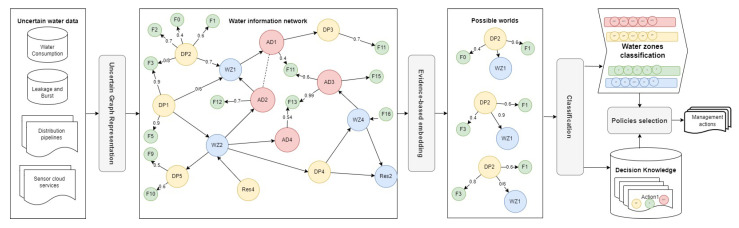
Evidence-based and embedding-driven classification of the water information network.

The water information network is first populated, then updated, by considering the new features of each water zone. The updated WIN in Figure 2 shows that the probability values of different features (KPI metrics) are represented by green nodes, while the weighted relations represent the probability associated with each KPI feature, such as pressure and pH. The representation of highly uncertain water environments will facilitate and accelerate the selection of corrective actions. That is achieved by adopting an uncertain classification of the WIN nodes with similar features/states (e.g., water zones with poor quality), as we will demonstrate in the next section.

Table 2 summarizes the basic symbols and notations used in the rest of this paper.

### 3.2. Uncertain Embedding of the Water Network

In our previous work, we proposed an embedding graph model that reduces the complexity of querying the water information network. This task consists of first locating the captured events (e.g., pollution, leakage, pressure loss), then evaluating and selecting the suitable management policy. The proposed embedding model maps the water information network into a set of vectors, each denoting a learned representation of water-related entities. The ones with similar features (e.g., reservoirs containing low-quality water) are mapped closer and classified together. However, the previous embedding model deals with valid facts only, which means it cannot handle uncertain facts (e.g., <*Pollution, ManagedBy, SedimentRemoval, 0.661*>) or estimate the confidence of unseen facts, i.e., latent relations.

**Definition** **5.**  *(Uncertain embedding) Given a water information network G, the uncertain embedding consists of encoding each entity v∈V and relation r∈E into a low-dimensional vector space while preserving not only the structural graph information, but also the confidence scores of the different relations. The uncertain embedding also aims at predicting the confidence score of latent connections between entities (e.g., <PressureLoss, ManagedBy, RestorePressure*, ?*>). Based on that, the proximity among the water network’s entities is preserved in the original UWIN.*

(3) $$v_{i}=\arg\min_{v\in R^{k}}\left|\right|f_{i}-{Wv||}_{2}^{2}+{\lambda ||v||}_{2}^{2}$$

Using a linear regression model, Equation (3) computes the vector representation $v_{i}$ of a data point $d_{i}$. $f_{i}$ denotes the feature vector of $d_{i}$, $W\in R^{m\times k}$ is the weight matrix to be learned, λ is a regularization parameter, and $\left|\right|\cdot {\left|\right|}_{2}$ is the L2-norm.

(4) $$P\left(e_{ij}\right)=\frac{1}{1+\exp(-\gamma_{0}w_{ij}+\gamma_{1})}$$

Equation (4) computes the probability $P(e_{ij})$ of an edge $e_{ij}$ being present between nodes $n_{i}$ and $n_{j}$. $w_{ij}$ denotes the weight of the edge, and $\gamma_{0}$ and $\gamma_{1}$ are hyperparameters to be learned.

(5) $$U\left(e_{ij}\right)=\frac{1}{1+\exp(-\gamma_{2}w_{ij}+\gamma_{3})}$$

Equation (5) computes the uncertainty $U(e_{ij})$ of an edge $e_{ij}$ in the uncertain information network G. $w_{ij}$ denotes the weight of the edge, and $\gamma_{2}$ and $\gamma_{3}$ are hyperparameters to be learned. The uncertainty is modeled as a logistic function of the edge weight.

The uncertain knowledge graph embedding (UKGE) method assigns a probability distribution to each entity and relationship in the knowledge graph, indicating the uncertainty of their actual embedding in the latent space. In Algorithm 1, this method is used to infer additional knowledge, such as latent connections, by generating a set of probability values that reflect the probabilistic distribution of the water network entities and their relationships. The probabilistic technique was chosen due to its widespread use in handling incomplete or uncertain data, as demonstrated in [10]. The following arguments justify our decision to use this approach:

- Firstly, it can help in quantifying the degree of uncertainty associated with the data collected from various sensors in the network. This can enable decision makers to have a more accurate understanding of the reliability of the data and, consequently, make more informed decisions.
- Secondly, probabilistic techniques can enable the representation of complex dependencies and correlations between the different factors that contribute to the uncertainty in the water zone data. This can help in building more accurate models that can better capture the underlying dynamics of the system and, in turn, improve the decision-making process.
- Finally, probabilistic techniques can provide a principled way of combining different sources of information, including historical data and expert knowledge, to arrive at a more comprehensive and robust assessment of the uncertainties in the water zones. This can lead to better-informed decisions that take into account a wide range of factors and sources of uncertainty.

**Algorithm 1:** Uncertain knowledge graph embedding for water quality management**Require:**    Water Quality Dataset $D=d_{1},d_{2},\ldots ,d_{n}$   Domain ontology O   Distance metric dist   Number of dimensions *k*   Hyperparameters: α, β, γ**Ensure:**    Uncertain Water Information Network G=(V,E,P)1:Initialize node set V={}2:Initialize edge set E={}3:Initialize probability set P={}4:**for **$d_{i}\in D$**do**5:      Extract features $f_{i}$ from $d_{i}$ using ontology O6:      Compute the vector representation $v_{i}$ of $d_{i}$ using Equation (3)7:      Create a node $n_{i}$ in V with attributes $f_{i}$ and embedding $v_{i}$8:      **for** $n_{j}\in V$ **do**9:          Compute the distance $d_{ij}=dist\left(v_{i},v_{j}\right)$ between node $n_{i}$ and $n_{j}$10:          **if** $d_{ij}\leq \alpha$ **then**11:             Create an edge $e_{ij}$ between $n_{i}$ and $n_{j}$ with weight $d_{ij}$12:             Set the probability $P(e_{ij})$ to β using Equation (4)13:          **end if**14:      **end for**15:**end for**16:**for **$e_{ij}\in E$**do**17:      Compute the uncertainty $U(e_{ij})$ using Equation (5)18:      Set the probability $P(e_{ij})$ to $\gamma \cdot U\left(e_{ij}\right)+\left(1-\gamma \right)\cdot P\left(e_{ij}\right)$19:**end for**

### 3.3. Uncertainty-Aware Decision Making for Water Management

In this section, we define an algorithm for querying the uncertain water information network to locate the affected water entities (e.g., low-quality reservoirs) and determine the most relevant management plan. The decision process is conducted under uncertainty of the monitored data and the learned representations, particularly the candidate management policies. This uncertainty varies depending on the water’s operational parameters (e.g., pH, turbidity, dissolved oxygen, rainfall, organic carbon, chemical dosage, flow rate, conductivity, disinfectant residual, and hydraulic pressure).

The example in Table 3 depicts a set of candidate management plans with their confidence scores. These are computed based on the uncertainty degrees quantified after the monitoring phase. For example, the *pressure loss* could be resolved by *flushing* or *disinfecting* the concerned water zone. Since flushing has a higher confidence (0.91) than disinfection (0.83), it will be selected by Algorithm 2.
**Algorithm 2:** Smart water decision making1:**Input: **W—Uncertain Water network, $L_{p}$—captured events.2:**Output: ***P*—water management plan.3:**Begin**4:P←⌀5:**for each **$e\in L_{p}$**do**6:      Locate *e* in W7:    **for each** action a∈Context(e)**do**  ▹ Obtain management actions for the affected water entity (event *e*)8:          **if** (e,managedBy,a)∈W**then**▹ Check the existence of the management action *a* in W9:              $l_{ea}\leftarrow Confidence\left(e,managedBy,a\right)$10:             $P\left[e\right]\leftarrow P\left[e\right]\cup \left(a,l_{ea}\right)$  ▹ Save corrective measure *a* for detected event *e*11:        **end if**12:   **end for**13:   Sort P[e] ▹ Sort candidate actions for event *e* according to their confidence score.14:**end for**15:**Return*** P*   ▹ Return water management plan with several alternatives

Algorithm 2 takes, as input, a set $L_{p}$ of captured deviations (e.g., pressure loss, pollution), in addition to the uncertain water information network W. The output is a set of actions denoting the water management plan with the highest confidence score. Each entity may be labeled with one or more events (e.g., pressure loss, chlorination, low nitrites level). Labeling water-related entities in the WIN allows arranging into groups of water zones that share similar captured changes. This classification enables smart management at the class level rather than triggering a management plan for each separate water zone.

For each node ($e\in L_{p}$) denoting the captured events in the water environment, Algorithm 2 starts by locating its connected actions (Context(e)), which represent the corrective measures to deal with *e* (line 6). Then, for each action *a*, the algorithm checks the existence of a valid triple in the possible world W⊆G (line 8). This step is essential, as a triple’s confidence score reflects its ability to solve the captured event *e* (line 9). In this case, the confidence score keeps or excludes a candidate management action (line 10). The event processing ends with the saving (line 10) and sorting (line 12) of the candidate’s actions. This routine is repeated for each captured event (line 5). It should be noted that the processed event concerns at least one water entity or a group, i.e., class, of entities that encounter the same deviation.

The complexity of Algorithm 2 mainly depends on the number of affected zones, i.e., captured events ($|L_{p}|$), and the UWIN size, i.e., number of triples (|W|). The cost of locating those events and determining each one’s candidate actions takes $O(|L_{p}|.|N\left(e\right)|)$, where N(e) is the context of an event *e*. For each potential management action a∈Context(e), Algorithm 2 checks the existence of a valid triple relating an occurring event *e* and the action *a*. After sorting the candidate actions, this operation takes O(|N(e)|.|P|). The whole time complexity is in $O(|L_{p}|.|N\left(e\right)|.|P|)$, and could be simplified to $O(|L_{p}{|}^{2}.|N\left(e\right)|)$, since the set *P* reflects the number of captured events.

## 4. Experiments

This section provides a detailed description of the data used in this study and the experimental setup. This includes information on the data sources, the preprocessing steps applied, and the evaluation metrics used to assess the performance of the proposed approach. This section also presents the study’s findings, including the impact of confidence levels on the accuracy of water zone classification. It provides a visualization of water zones embedding, which can aid in decision-making related to water management.

In this study, we developed the solution to encode the whole water management process (implementation source code and configuration information are available at https://github.com/msellamiTN/ukge-smartwater2022, accessed on 7 May 2023). We used the TensorFlow [40] and scikit-learn libraries [41] to encode the entire water management process. The t-distributed stochastic neighbor embedding library (t-SNE) [42] was used to project and visualize the water environment data and reduce their dimensionality.

### 4.1. Dataset and Experimental Protocol

We utilized a publicly available dataset called “Indian water quality data” that encompasses historical water quality information from specific locations in India [43]. This dataset includes measurements of pollutants, which are recorded as average values over a certain period. The data were sourced from official websites maintained by the Indian government. The physicochemical characteristics that describe each sample in the dataset are as follows:

1. Temperature: The temperature of water samples can affect various physical and chemical properties, such as the density, viscosity, and solubility of different substances.
2. pH: The pH level of water samples indicates their acidity or alkalinity, which can affect the chemical reactions and the behavior of different substances in water. The pH scale ranges from 0 to 14, with 7 being considered neutral, below 7 acidic, and above 7 alkaline or basic.
3. Conductivity: Conductivity is a measure of the ability of water to conduct electric current, which is influenced by the presence of dissolved ions or salts.
4. Dissolved oxygen (DO): DO is the amount of oxygen dissolved in water, which is critical for the survival of aquatic organisms and the health of aquatic ecosystems.
5. Biological oxygen demand (BOD): The amount of oxygen required by microorganisms to break down organic matter in the water sample, measured in milligrams per liter (mg/L).
6. Nitrate (NI): The concentration of nitrate ions in the water sample, usually measured in milligrams per liter (mg/L).
7. Fecal coliforms (FC): The presence or concentration of fecal coliform bacteria in the water sample, often used to indicate fecal contamination and potential health risks.
8. Total coliforms (TC): The presence or concentration of total coliform bacteria in the water sample, including fecal and non-fecal coliforms.

As the dataset lacked information on triggering events and their accompanying circumstances, the Water Quality Index (WQI) was computed for each sample using Equation (6) and used to categorize water samples. The WQI is computed as the weighted sum of the quality rating scale of the parameters, where the weights are determined by the unit weight of each parameter, calculated using Equations (6). Here, *N* represents the total number of parameters used to calculate the WQI, and wj is the unit weight of the parameters used [24,44].

(6) $$WQI=\frac{\sum_{j=1}^{N}q_{j}*w_{j}}{\sum_{j=1}^{N}w_{j}}$$

### 4.2. Experimental Results

To examine the performance of our proposed approach, we performed various experiments, which are mainly related to the effect of uncertainty. In the first experiment, we studied how confidence levels affect the accuracy of water zones’ classification and, subsequently, the selection of water management policies. In the second experiment, we analyzed the effect of uncertainty in high- and low-confidence settings to uncover all unclassified water areas. This allowed us to gain a deeper understanding of the significance of accounting for uncertainty in different scenarios to improve the quality of water area classification.

#### 4.2.1. Impact of Confidence on the Accuracy of Water Zones’ Classification

**Figure 3:**
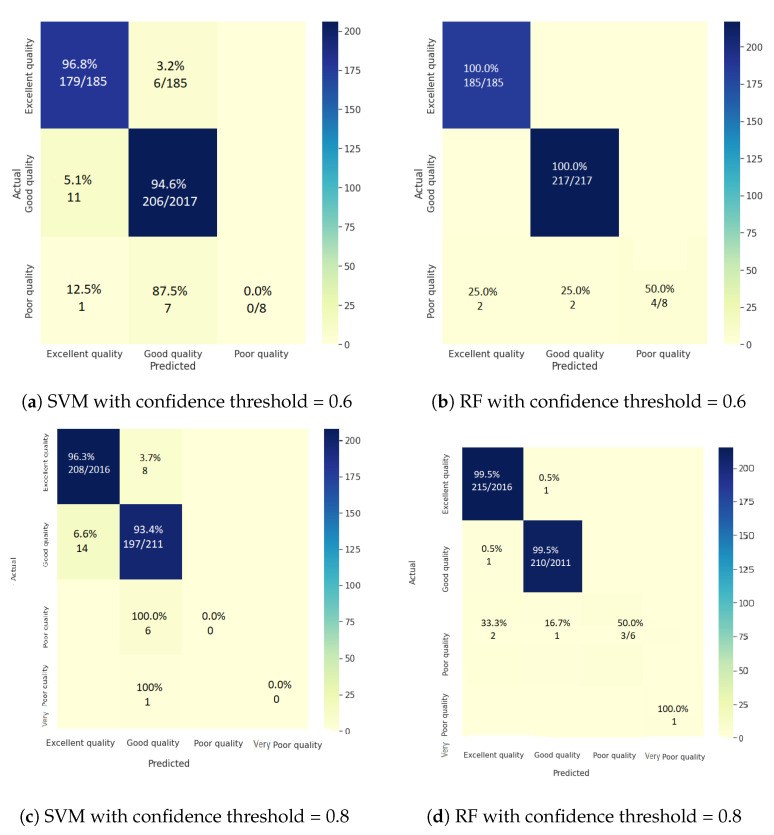
Normalized confusion matrices for the water zones’ embedding classification.

In these experiments, we studied the impact of varying the threshold between 0.6 and 0.8 on the accuracy of the water zones’ embedding classification. Figure 3 shows the confusion matrices of the two classifiers, SVM and RF, according to the four classes (excellent, good, poor, and very poor).

From Figure 3, we can see that using high confidence UKGE improves the classification performance of all classifiers by at least 7%. These findings highlight the importance of uncertainty in achieving accurate water zone classification based on sensor data. Indeed, the consideration of uncertain knowledge can help in the learning of appropriate water information network representations. UKGE-learned embeddings effectively capture uncertain information and constantly outperform the SVM classifier under high and low uncertainty scores, yielding promising outcomes with the RF classifier.

**Figure 4:**
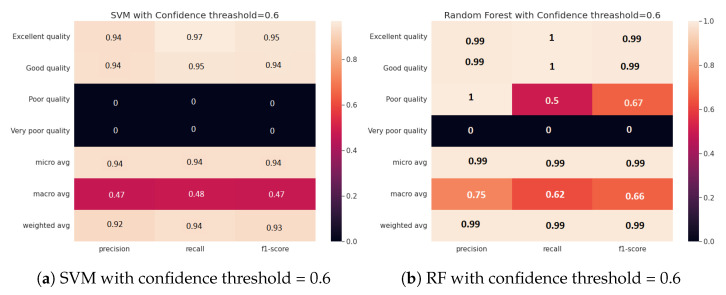
Normalized confusion matrices for the water zones’ embedding classification.

Figure 4 demonstrates how confidence impacts water classification performance, notably for the SVM classifier, which experiences an 8% decrease in accuracy at low confidence, probably resulting in unclassified water zones. The RF classifier, on the other hand, is less affected by low confidence, with just a 2% decrease in accuracy. This emphasizes the significance of monitoring data accuracy in the water classification process and establishing an appropriate confidence threshold depending on the chosen classifier to ensure feasible management policies.

**Figure 5:**
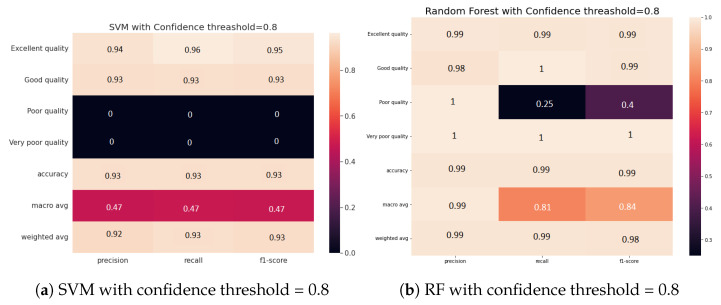
Normalized confusion matrices for the water zones’ embedding classification.

Furthermore, the results presented in Figure 5 imply the effectiveness of the proposed approach for classifying uncertain water zones, particularly those with very poor quality. This is demonstrated by the meaningful increase in classification accuracy from 0% to 100% when high confidence scores are considered. On the other hand, when the monitoring data are not certain (i.e., low confidence score), the embedding model may fail to recognize certain water zones, leading to lower accuracy in the classification process.

**Figure 6:**
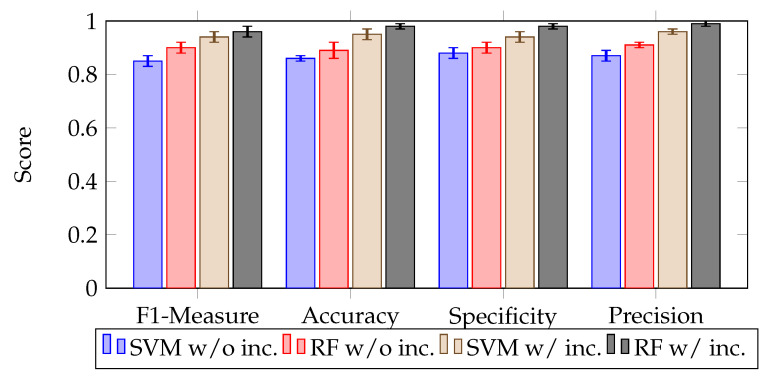
Comparison of the classification quality with and without uncertain embedding using SVM and random forest for different metrics.

Figure 6 presents classification performance measures with and without uncertain graph embedding, including F1 measure, accuracy, specificity, and precision. The findings show that including uncertain graph embedding improves classification quality significantly for both SVM and RF classifiers compared to the approach that considers only precise water data. RF surpasses SVM in all measures, with and without uncertainty consideration, particularly in accuracy and F1. This leads us to conclude that utilizing uncertain graph embeddings can effectively improve the accuracy of water zones’ classification. Additionally, we can deduce that RF performs better than SVM in the embedding classification task. We also observed that adjusting the confidence threshold can help in identifying low-quality areas, which can be undetected due to the dynamics of the water environment. Finally, we emphasize that selecting the effective classifier is a critical factor that impacts the classification performance, and this decision should be made based on the desired confidence level.

#### 4.2.2. Water Zones Embedding Visualization

**Figure 7:**
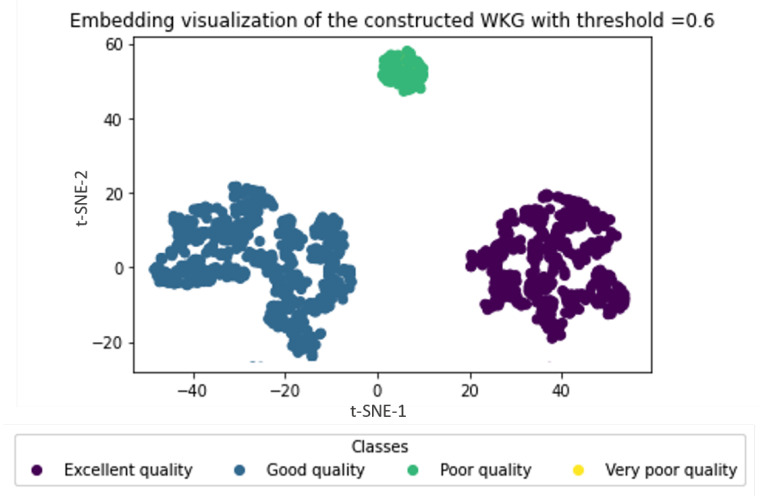
Embedding visualization of the constructed WKG with threshold = 0.6.

**Figure 8:**
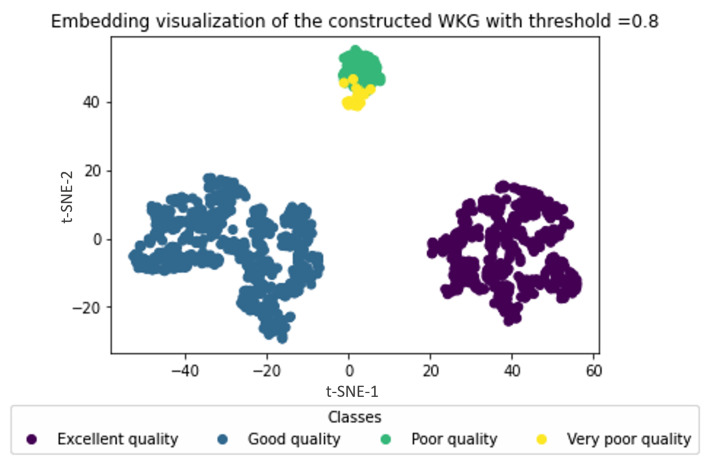
Embedding visualization of the constructed WKG with threshold = 0.8.

In these experiments, we varied the confidence threshold and analyzed its impact on the uncertain water graph embedding process. The results are recorded in Figure 7 and Figure 8.

Figure 7 shows that several water zones cannot be identified with low confidence. This implied that low-confidence zones had been neglected during the embedding process. For instance, with confidence of less than 0.6, water zones with very low quality have been excluded from the water zone classification process. These outcomes clearly reflect the importance of the confidence threshold and the water data uncertainty handling during the data analysis and embedding process. Water zones with low confidence should not be disregarded, but rather treated appropriately to ensure the accuracy and quality of the decision process. In addition, these results can be used to optimize water zone classification and improve the selection of water management policies.

Figure 8 also demonstrates that impoverished quality water zones were detected with a high confidence of 0.8. Thus, it can be concluded that embedding the uncertain graph enhances the classification of water zones by revealing the water zones with high uncertainty. This feature is crucial in highly dynamic and smart environments. By varying the confidence threshold, the water zone classification process can significantly improve the accuracy of decisions produced by the water management system. Thus, it is essential to determine the appropriate confidence threshold that aligns with environmental policies and requirements to obtain the best results for water zones’ classification and monitoring in smart environments.

Summarizing the above results, it was proven that handling the uncertainty in the water information network had positively impacted the recommendation of the appropriate water management actions. The embedding-driven classification of water zones depending on their current state helped arrange water zones according to their quality level. This arrangement was considerably improved with the incorporation of uncertainty factors. For instance, low-confidence water zones (i.e., high uncertainty) were excluded from the management process to avoid inappropriate recommendations. In this way, the decision on the water zones’ quality (excellent, good, poor, very poor) is based on a strictly refined set of classes. Contrariwise, higher confidence scores have increased the likelihood of accurately classifying a water zone into one of the considered quality levels. That is understandable because the high confidence score transformed the water information network into a deterministic one, thus correctly treating this content in its vectorized form.

In this study, we proposed an approach for decision making in IoT-based water environments through probabilistic and evidence theory based knowledge graph embedding. However, several limitations need to be addressed. These limitations include the following:

- Handling different types of uncertainty: The use of other techniques for modeling uncertainty, such as fuzzy logic systems and possibility-based theory, can help handle uncertainty in water environments, which is crucial for making accurate and reliable decisions.
- Improving network representation learning: While knowledge graph embedding is a powerful technique, there are other network representation learning techniques, such as graph convolutional networks and attention-based models, that can potentially provide more accurate and informative embeddings of water entities.
- Distributed learning: The application of the distributed learning concept to water networks can enable collaborative, scalable, and privacy-preserved analytics of water data in larger-scale and more complex smart water networks, leading to better decision making and resource management.

## 5. Conclusions

This work focuses on managing smart water environments by proposing an uncertainty-aware decision support system that uses data collected by a network of sensors. The system leverages probabilistic techniques and network representation learning to create a probabilistic embedding of the water information network entities. The uncertain representations are classified using network representation learning, and evidence theory was applied to make decisions aware of the sensed water data uncertainties. The proposed system triggers appropriate water management policies, considering the incompleteness and imprecision of the sensed water data. The experimental results have proven the effectiveness of our approach in handling uncertainty in the vectorized water network.

As future research directions, we intend to use advanced probabilistic models to handle uncertainty in the water information network, such as fuzzy logic systems and possibility theory. We also will investigate the use of other network representation learning techniques (e.g., graph convolutional networks and attention-based models) to learn more accurate and informative embeddings of water entities. Additional management capabilities will also be incorporated into the proposed decision support system to handle other water-related problems (e.g., water resource allocation, water pollution detection, and groundwater depletion). Finally, a federated learning approach is underway to ensure collaborative, scalable, and privacy-preserving water data analytics in larger scale and more complex smart water networks.

## Figures and Tables

**Table 1:** Comparison between existing water management solutions and our approach.

Comparison Criteria	Existing Approaches	Our Approach
Management granularity	Entity level	Class level
Uncertainty handling	No	Yes
Monitoring data	Static	Dynamic
Monitoring space	Not specified	Heterogeneous water zones
Others	⊖ Data impreciseness ⊖ No uncertainty quantification ⊖ Decision complexity ⊖ No unified representation ⊖ No corrective actions ⊖ No consideration of temporal variations	⊕ Low number of management policies ⊕ Reduced decision time ⊕ Fewer policy conflicts ⊕ Accurate decision ⊕ Improved capabilities for corrective action suggestions ⊕ Improved capabilities for temporal variation consideration

**Table 2:** Basic symbols and notations.

Symbol	Definition
G	Uncertain Water Information Network (UWIN)
$G_{w}\subseteq G$	A snapshot, i.e., possible world of the water network, given the monitored data
E	Set of connections between UWIN entities
$E_{w}\subseteq E$	Set of valid relations in the Water Information Network W
$\left(e_{i},r,e_{j},l\right)$	An uncertain fact in G
$\left(e_{i},r,e_{j}\right)$	A valid fact in W
w,p,f	Embeddings of water entities, management policies, and feature entities, respectively,
*d*	The dimension of embeddings
$R^{d}$	*d*-dimensional continuous vector space
$v^{w}$, $v^{p}$, $v^{f}$, $v^{r}$	Vector representations of entities (*w*,*p*,*f*) and relations (*r*) in the UWIN.
$D^{+}$, $D^{-}$	Sets of positive and negative triples
L	A function denoting the objective loss function for the uncertain embedding

**Table 3:** Example of corrective measures with their triggering probability (confidence score).

Event	Corrective Measure	Confidence
Turbidity [>1 NTU]	Settling and decanting	0.78
Pressure loss [<20 psi]	Flushing	0.91
Pressure loss [<20 psi] OR Pumps fail	Disinfection	0.83
Pollution	Sediment removal	0.95

## Data Availability

Not applicable.
